# A Novel Digital Health Platform With Health Coaches to Optimize Surgical Patients: Feasibility Study at a Large Academic Health System

**DOI:** 10.2196/52125

**Published:** 2024-04-04

**Authors:** Stephen Andrew Esper, Jennifer Holder-Murray, Katie Ann Meister, Hsing-Hua Sylvia Lin, David Kojo Hamilton, Yram Jan Groff, Brian Scott Zuckerbraun, Aman Mahajan

**Affiliations:** 1 Department of Anesthesiology and Perioperative Medicine University of Pittsburgh Medical Center Pittsburgh, PA United States; 2 Department of Surgery University of Pittsburgh Medical Center Pittsburgh, PA United States; 3 Department of Anesthesiology and Perioperative Medicine University of Pittsburgh Pittsburgh, PA United States; 4 Department of Neurosurgical Surgery University of Pittsburgh Medical Center Pittsburgh, PA United States; 5 Department of Orthopedic Surgery University of Pittsburgh School of Medicine Pittsburgh, PA United States

**Keywords:** digital health solution, feasibility, length of stay reduction, patient engagement, patient satisfaction, perioperative medicine

## Abstract

**Background:**

Pip is a novel digital health platform (DHP) that combines human health coaches (HCs) and technology with patient-facing content. This combination has not been studied in perioperative surgical optimization.

**Objective:**

This study’s aim was to test the feasibility of the Pip platform for deploying perioperative, digital, patient-facing optimization guidelines to elective surgical patients, assisted by an HC, at predefined intervals in the perioperative journey.

**Methods:**

We conducted an institutional review board–approved, descriptive, prospective feasibility study of patients scheduled for elective surgery and invited to enroll in Pip from 2.5 to 4 weeks preoperatively through 4 weeks postoperatively at an academic medical center between November 22, 2022, and March 27, 2023. Descriptive primary end points were patient-reported outcomes, including patient satisfaction and engagement, and Pip HC evaluations. Secondary end points included mean or median length of stay (LOS), readmission at 7 and 30 days, and emergency department use within 30 days. Secondary end points were compared between patients who received Pip versus patients who did not receive Pip using stabilized inverse probability of treatment weighting.

**Results:**

A total of 283 patients were invited, of whom 172 (60.8%) enrolled in Pip. Of these, 80.2% (138/172) patients had ≥1 HC session and proceeded to surgery, and 70.3% (97/138) of the enrolled patients engaged with Pip postoperatively. The mean engagement began 27 days before surgery. Pip demonstrated an 82% weekly engagement rate with HCs. Patients attended an average of 6.7 HC sessions. Of those patients that completed surveys (95/138, 68.8%), high satisfaction scores were recorded (mean 4.8/5; n=95). Patients strongly agreed that HCs helped them throughout the perioperative process (mean 4.97/5; n=33). The average net promoter score was 9.7 out of 10. A total of 268 patients in the non-Pip group and 128 patients in the Pip group had appropriate overlapping distributions of stabilized inverse probability of treatment weighting for the analytic sample. The Pip cohort was associated with LOS reduction when compared to the non-Pip cohort (mean 2.4 vs 3.1 days; median 1.9, IQR 1.0-3.1 vs median 3.0, IQR 1.1-3.9 days; mean ratio 0.76; 95% CI 0.62-0.93; *P*=.009). The Pip cohort experienced a 49% lower risk of 7-day readmission (relative risk [RR] 0.51, 95% CI 0.11-2.31; *P*=.38) and a 17% lower risk of 30-day readmission (RR 0.83, 95% CI 0.30-2.31; *P*=.73), though these did not reach statistical significance. Both cohorts had similar 30-day emergency department returns (RR 1.06, 95% CI 0.56-2.01, *P*=.85).

**Conclusions:**

Pip is a novel mobile DHP combining human HCs and perioperative optimization content that is feasible to engage patients in their perioperative journey and is associated with reduced hospital LOS. Further studies assessing the impact on clinical and patient-reported outcomes from the use of Pip or similar DHPs HC combinations during the perioperative journey are required.

## Introduction

The annual surgical volume in the United States is estimated at 48.4 million procedures [1]. Though heart disease and stroke may be the 2 leading causes of worldwide mortality (25% or 15 million deaths) [2,3], before the SARS-CoV-2 pandemic, postoperative surgical mortality was the third leading contributor to death in the United States [2]. Furthermore, the occurrence of postoperative 30-day complications is expected to rise to 15% among all patients and cost over US $11,000 per case, or US $31.35 billion nationally, on an annual basis [4,5]. Improving surgical quality of care to reduce mortality, complications, readmissions, and emergency department (ED) visits represents an enormous opportunity for the health care system. To reduce surgical complications and improve postoperative outcomes, focus has shifted to optimizing patients preoperatively and postoperatively through strategies such as prehabilitation, improvement in medical comorbidity, and enhanced recovery after surgery protocols [6]. Because mobile and wireless technologies have become increasingly accessible and capable on a global scale [7], digitization of protocols and other health interventions is being developed as a means to improve quality of care while reducing cost.

The field of digital health has grown over the past several years with advances in digital health platforms (DHPs) or telemedicine services, which have allowed deployment in select patient populations to improve chronic health conditions [8]. Several mobile apps have been developed and used as tools to help provide perioperative instructions as well as protocol guidance for patients. Feasibility studies have shown these DHP are convenient for patients to use in orthopedic surgery [9,10] and gastrointestinal surgery [11-13]. Yet, outcomes results have been mixed [14,15] or not yet studied to date. Furthermore, the DHP content is often narrow and applied to one surgery type or a specific problem, such as activity or pain management, rather than more holistic prehabilitation and curated to each patient’s needs based on patient comorbidity, activity level or ability, or nutritional status. Additionally, these DHPs did not use a one-on-one health coach (HC) in addition to the DHP to assist patients in achieving their goals. Finally, there continues to be a significant unmet need within health care to provide patients undergoing surgery with high-quality education, optimization, and care coordination throughout the complex preoperative and postoperative journey. Our hospital desired to pilot an integrated DHP with human digital HCs to improve patient preoperative optimization, surgical care coordination, and outcomes. To address this need, we partnered with a novel perioperative DHP company, Pip Care, to create digitized perioperative patient-facing optimization guidelines and surgical instructions for our surgical population. Pip simplifies the patient’s health care plan into definable, easy-to-understand, and complete daily tasks and uses regular HC contact to improve outcomes, thus setting Pip apart from other DHPs. The aim of this study was to test the feasibility and acceptability of the novel Pip platform in deploying perioperative patient-facing optimization guidelines to elective surgical patients both digitally and with the assistance of an HC at predefined intervals in the perioperative journey and to report clinical outcomes and patient satisfaction with the use of Pip.

## Methods

### Overview

We partnered with Pip Care to develop perioperative content and test the deployment of Pip perioperatively. Pip is a HIPAA (Health Insurance Portability and Accountability Act)-compliant, personalized, and interactive DHP that functions on both iOS (Apple Inc) and Android (Google Inc) operating systems.

### Design of Clinical Pathways and Pip Functionality

A multidisciplinary team in perioperative care at our academic medical center from anesthesiology, surgery, and nursing defined the pathway content and patient tasks to be digitized on the Pip platform. These perioperative clinical pathways included preoperative nutrition, preoperative fitness, smoking cessation, preparation for surgery, day-before surgery planning, home preparation, and recovery after surgery. Patients were digitally assigned the appropriate clinical pathways by the human HC following the initial HC-patient intake and the HC’s review of the patient’s comorbidities from the electronic medical record. The tasks were prompted to the patient at appropriate intervals. Certified human HCs employed by Pip Care received education regarding the clinical pathways and were trained to interact with the electronic medical record for data collection and communication. HCs were responsible for motivating patients to reach their pre- and postsurgery goals through at least weekly one-on-one video or audio sessions; during these sessions, HCs would also answer any questions, provide educational content, track patient-reported outcomes (PROs), communicate patient progress to the provider, and facilitate referrals and resources if needed, in coordination with the perioperative clinical team members (Table 1). In addition to HC follow-up, patients were invited to explore a host of educational multimedia resources on disease processes and why optimization of said diseases is important before surgery.

**Figure 1:**
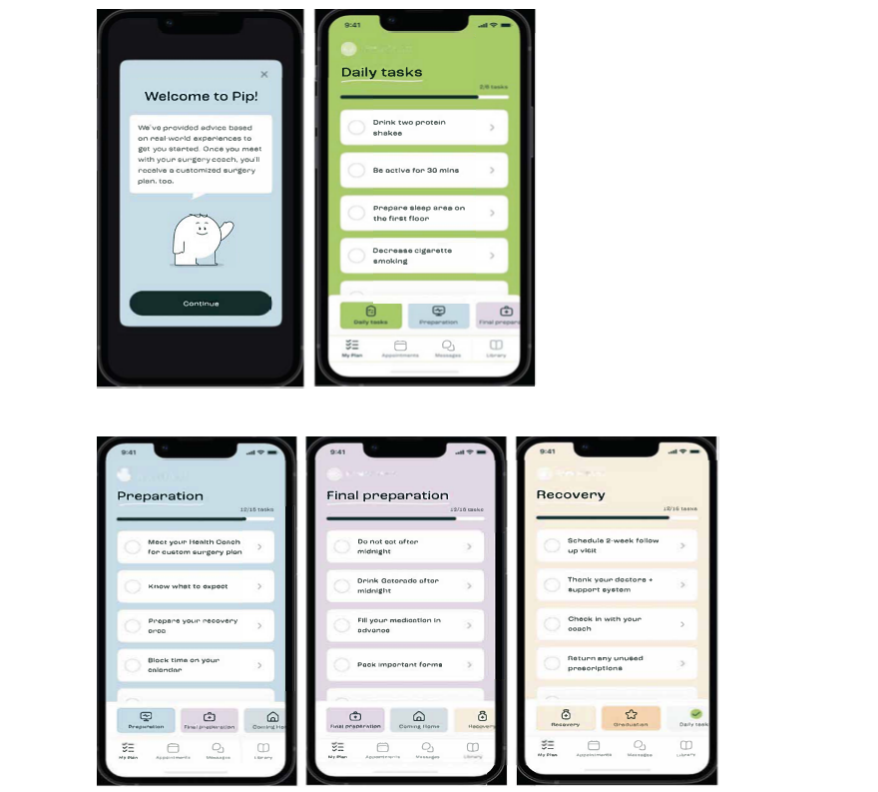
Screenshots of Pip with personalized protocols and daily tasks in My Plan.

**Figure 2:**
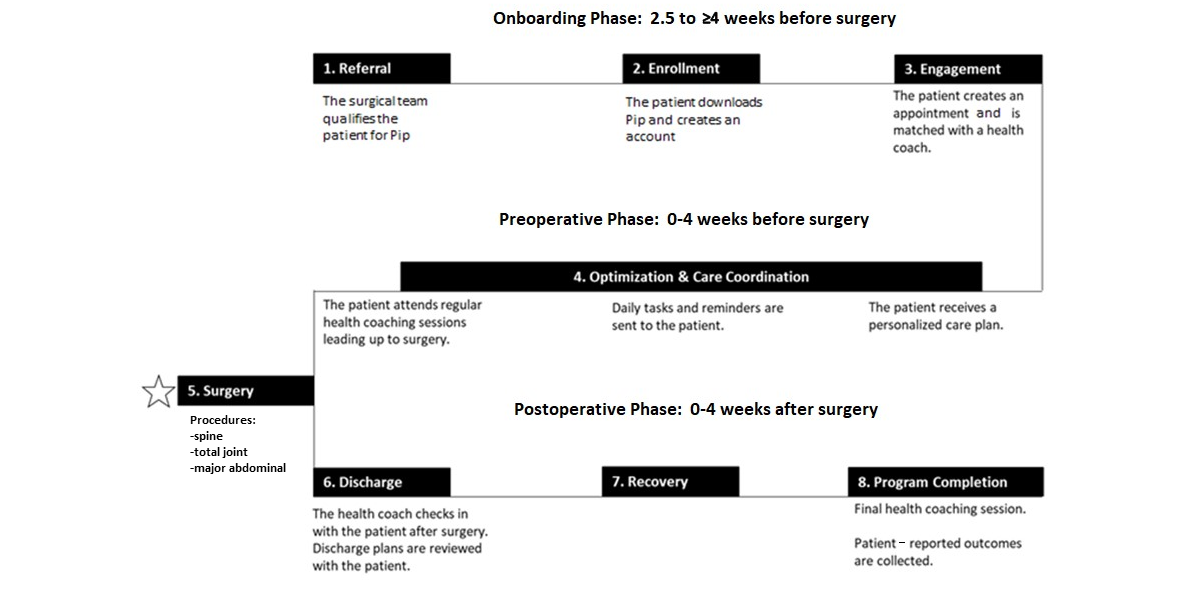
Pip patient engagement map. HC: health coach.

**Figure 3:**
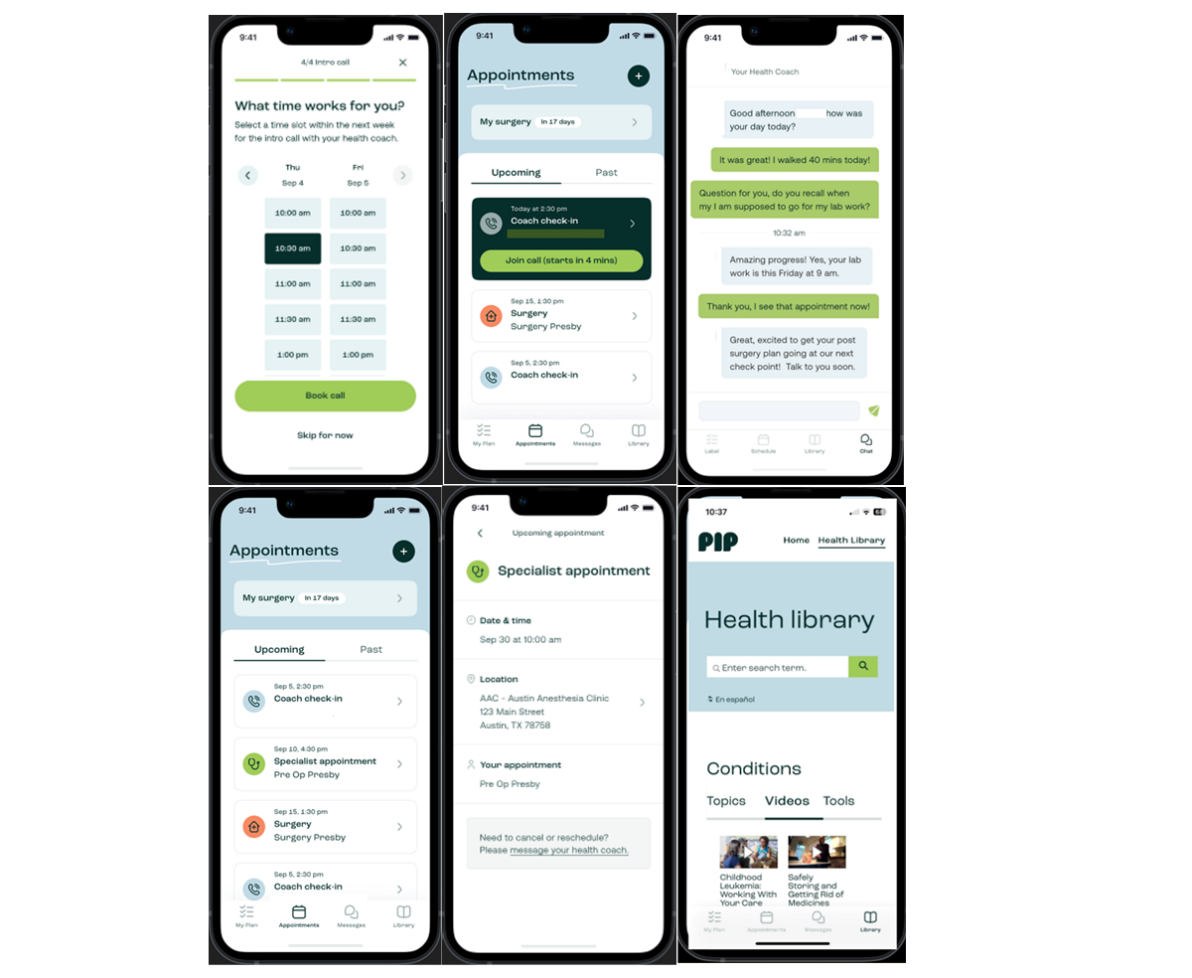
Screenshot examples of the Pip Appointments section, messaging, and entry into the Pip Library.

Pip contains 4 key features, which are represented by separate pages within the DHP user experience. The first is Pip My Plan, which displays the assigned personalized care plans and tasks by their HC (Figure 1). The second is Pip Appointments. Patients were asked to schedule weekly digital HC sessions through the Appointments page. The HC also populated the patient’s surgery-related appointments into this section for easy patient viewing (Figure 2). Third, patients have access to unlimited engagement with their HC through the Pip Messages page (Figure 3). Finally, patients have further unlimited access to a library of health system-approved education content, including articles and videos, to assist with their surgery preparation and recovery (Figure 3).

**Table 1:** Pip health coach (HC) tasks.

Category and tasks			Task description
**Referral management**			
	Review the EMR^a^ Pip List for newly added patients.	HC reviews the EMR to identify and validate referred patients.	
	Referred patient data transfer	HC transcribes the appropriate patient information into the Pip database.	
**Pilot enrollment and activation**			
	Execute the enrollment conversion plan.	HC executes a time-cadence enrollment conversion plan until the patient has enrolled in the pilot study or until the enrollment conversion plan ends.	
	Execute the patient activation conversion plan.	HC executes a time-cadence activation conversion plan until the patient has scheduled an “Initial HC Session” or until the activation conversion plan ends.	
**Surgery coaching and care plan management**			
	Weekly health coaching sessions.	For an estimated 4 weeks before surgery and 4 weeks postsurgery, HC conducts 30-minute weekly coaching sessions with patients to assist with surgery preparation and recovery.	
	Coaching session documentation	HC documents “encounter notes” from each coaching session.	
	Coaching session scheduling	HC schedules the subsequent coaching session.	
	Midweek patient check-in	In between weekly sessions, the HC sends at least 1 message (in-app or SMS text message) to the patient.	
	Patient communication through the in-app message	HC responds to the patients’ messages when they are received.	
	Distribution of surgery-related educational materials	HC sends patients applicable educational content on best practices for surgery preparation and recovery.	
	Patient care plan assignment and management	HC assigns and manages the patient’s care plans, including fitness, nutrition, smoking cessation, and discharge planning.	
**Provider communication**			
	Weekly patient progress report sent through EMR Encounter Note	HC’s encounter note in the EMR is sent to the clinical provider, detailing the patient’s status and adherence to protocols.	
	EMR InBasket communication	When an HC receives an out-of-scope question from a patient or learns of an escalated clinical issue, the HC messages the provider through EMR InBasket to escalate the clinical issue.	
	Provider synchronization calls	HC participates in daily and weekly synchronization calls with the provider team to ensure good communication and proper workflows.	
**Care coordination**			
	Surgery-related appointments	HC reviews the EMR and ensures all surgery-related clinical appointments are properly displayed within the Pip app. The HC encourages attendance at these appointments through messaging and during coaching sessions.	
	Facilitating health system resources for patients	HC facilitates health system-specific surgery-related resources for the patient as needed.	
**Patient-reported outcome and satisfaction data collection**			
	Collecting patient satisfaction surveys	HC sends an anonymous patient satisfaction survey to patients.	
	Collecting PROs^b^	HC collects PROs upon the patient’s completion of the pilot program.	
**Service recovery**			
	Digital platform trouble shooting	HC assists with any issues with the technology.	

^a^EMR: electronic medical record.

^b^PRO: patient-reported outcome.

### Feasibility Study

This study is an institutional review board/quality improvement review committee–approved (ID 3949) descriptive and prospective feasibility study of patients scheduled for elective abdominal, spine (cervical, lumbar, thoracic, and combined), and total joint replacement (hip or knee) surgery, invited to enroll in Pip from 2.5 to 4 weeks preoperatively through 4 weeks postoperatively at a single academic medical center from November 22, 2022, to March 27, 2023. Inclusion criteria were being aged 18 years or older; ability to speak and understand English; scheduled elective abdominal, spine, or joint replacement surgery; having more than 1 comorbidity linked to increased surgery risk (eg, type 2 diabetes, being aged 70 years or older, having a BMI greater than 40 kg/m^2^, high blood pressure, and smoking history); no recent hospitalization for medical comorbidity that may impact surgical timing, such as heart failure (in order to ensure surgical date was likely); daily access to a tablet or smartphone; and technological literacy (ability to navigate digital devices with oversight or perioperative team assistance). Exclusion criteria include surgery not scheduled, canceled or delayed, or a change in scheduled surgery type.

Patients were recruited continuously from our perioperative clinic until the desired pilot sample size of approximately 150 patients was reached. All patients received our standard perioperative risk assessment, optimization, and educational content from our perioperative clinic. After enrollment, patients downloaded and enrolled in Pip. Patients scheduled their first digital one-on-one session with the HC through the Pip platform after enrollment, and this first HC visit was typically scheduled within 1 week or less. HC visits were offered weekly preoperatively and weekly following hospital discharge. If at any time the patient desired to leave the study, they were able to withdraw (Figure 2).

The number of patients who were invited, enrolled, activated, and completed the program was collected. The number of health coaching sessions attended and the time from enrollment to surgery were collected. Patient-specific characteristics included age, institutional perioperative risk score (low being less than 2%, intermediate being between 2% and less than 5%, and high being 5% or more risk of mortality or major adverse cardiac or cerebrovascular events) [16], type of surgery, length of stay (LOS), readmission, and ED visits. Primary end points include patient satisfaction, patient engagement, and Pip HC evaluations. We used industry benchmarks to compare our DHP enrollment rate [17,18], surgery completion with enrollment [18], and postsurgery engagement rates [17,18]. Patients’ overall satisfaction was assessed by the topline patient satisfaction surveys with score ratings from 1 to 5, with 1 denoting the lowest satisfaction and 5 denoting the highest satisfaction [19]. Pip HCs were evaluated based on a scaled numerical response to the question, “How much do you agree with the statement: My Pip Health Coach Helped Me Prepare for and Recover from Surgery?” using a score rating scale of 1 to 5, with 1 denoting “strongly disagree” and 5 denoting “strongly agree.” The Pip experience was evaluated for acceptability using the net promoter score with the question, “Using a scale of 1 to 10, how likely are you to recommend Pip to a friend or colleague?” with 1 denoting “least likely” and 10 denoting “most likely.”

Secondary end points included LOS, 7- and 30-day readmission rates, and ED use within 30 days. In order to evaluate the effect of Pip, patients receiving the Pip program were compared with a non-Pip group of patients. This control group of patients included patients who were aged 18 years or older and underwent elective surgery of the same type from January 1, 2022, to December 31, 2022.

### Statistical Analysis

The primary end points are descriptive. Secondary end points required further statistical analysis. Continuous variables were summarized using the mean (SD) or median (IQR) when appropriate. Categorical variables were summarized by frequencies and percentages. The chi-square test was used for differences in proportions for categorical variables, and the Student *t* test or nonparametric Kruskal-Wallis test was used to determine the differences in the distribution of continuous data between the Pip and non-Pip groups. Stabilized inverse probability of treatment weighting (SIPTW) was created to reduce selection bias and balance the patient characteristics (ie, age, procedures, and perioperative risk score) in the Pip and non-Pip groups [20,21]. A marginal structural model with log-linked gamma distribution and SIPTW was used to estimate the mean ratios of the LOS between the Pip and non-Pip groups. Marginal structural models with log-binomial distribution and SIPTW were used to estimate the relative risk of 7-day hospital readmission, 30-day hospital readmission, and 30-day ED use [21]. All tests were two-sided and a *P* value of less than .05 was used to indicate statistical significance. SAS (version 9.4; SAS Institute) was used for statistical analyses.

### Ethical Considerations

The study was granted a waiver of consent as the risk to the patient was considered to be minimal and was considered to be a quality improvement study. All patient participation was voluntary, and no patient received compensation.

## Results

### Engagement Outcomes

**Figure 4:**
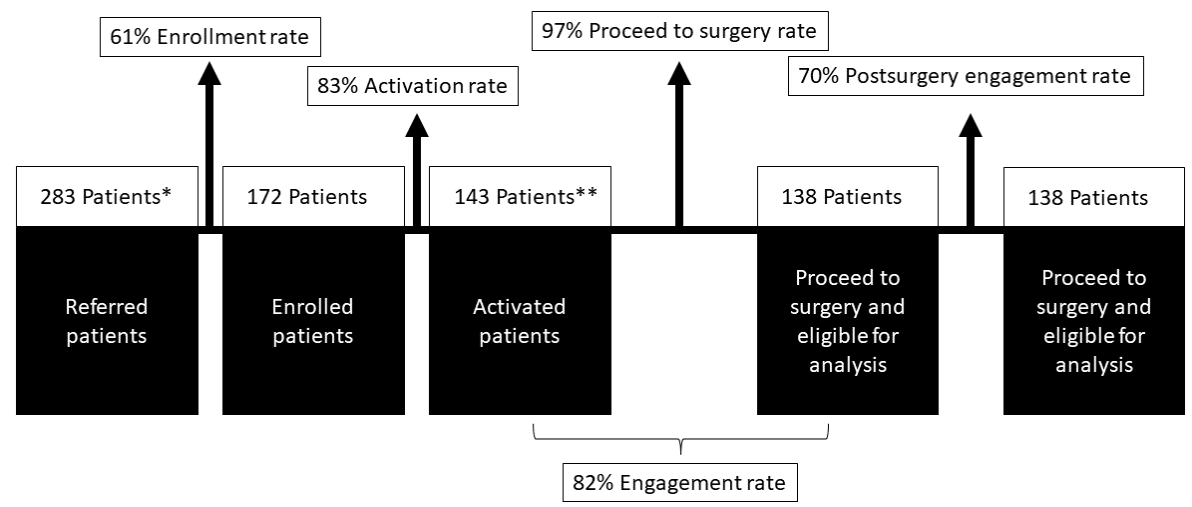
Pip patient engagement map. *Data set does not include 2 patient referrals who were out of scope of pilot parameters. ** Exclusions to analysis are as follows: patient did not need surgery (n=1); patients referred for alternative lower-risk therapy (n=2); surgery delayed and patient rescheduled (n=1); surgery delayed and Pip could not access the patient’s chart (n=1).

Out of 283 patients invited to participate in Pip, 172 (60.8%) were enrolled, compared to industry benchmarks (5%-30%). A total of 5 patients who enrolled were excluded from this analysis due to surgery delay, cancellation, or alternative surgery scheduled. Of those enrolled, 83.1% (143/172) had ≥1 HC session. Of the patients who had ≥1 HC session, 97.2% (138/142) proceeded to surgery, an improvement compared to industry benchmarks (90%-93%) [17,18]. After surgery, 70.3% (97/138) patients engaged with Pip postoperatively, compared to the industry benchmarks (31%-52%; Figure 4). Pip demonstrated an 82% weekly engagement rate, defined as repeat attendance at HC sessions. There was an average of 27 (range 7-108) days of lead time from enrollment to surgery, and patients attended an average of 6.7 (range 3-19) HC sessions. Pip received a total of 95 patient satisfaction survey submissions. Patients reported an overall high level of satisfaction based on the topline survey (mean 4.8/5; n=95; Table 2). Patients strongly agree that HC helped them throughout the perioperative process based on the Pip HC evaluation (mean 4.97/5; n=33). To measure acceptability, the net promotor score rating score was obtained; of the 33 respondents, the mean score was 9.7 out of 10.

**Table 2:** Aggregate patient satisfaction scores (average score rating was 4.8 out of 5).

Score	Surveys completed (n=95), n (%)
1	0 (0)
2	1 (1)
3	1 (1)
4	16 (17)
5	77 (81)

### Clinical Outcomes

There were a total of 367 patients in the non-Pip group and 138 patients in the Pip group. After creating SIPTW based on age, procedures, and perioperative risk score, a total of 268 patients in the non-Pip group and 128 patients in the Pip group had appropriate overlapping distributions of SIPTW for the analytic sample. Before SIPTW, age and preoperative risk score were shown to be significantly different between the Pip and non-Pip groups (Table S1 in Multimedia Appendix 1). Baseline patient characteristics weighted by SIPTW showed a balanced age, procedure type, and preoperative risk score between the 2 groups (Table 3). The Pip cohort was associated with both mean and median reductions in LOS when compared to the non-Pip cohort (mean 2.4 vs 3.1; median 1.9 IQR 1.0-3.1 vs median 3.0, IQR 1.1-3.9). Pip was significantly associated with a 24% reduction in postoperative LOS (mean ratio 0.76; 95% CI 0.62-0.93; *P*=.009 Table 4). Pip care was associated with a 49% lower risk of 7-day readmission (relative risk [RR] 0.51; 95% CI 0.11-2.31; *P*=0.38) and a 17% lower risk of 30-day readmission (RR 0.83; 95% CI 0.30-2.31; *P*=.73), though not statistically significant. Pip and non-Pip groups had similar risk in 30-day ED returns (RR 1.06; 95% CI 0.56-2.01; *P*=.85).

**Table 3:** Weighted patient characteristics by Pip versus non-Pip.

Variable			Non-Pip (n=268; 68%)		Pip (n=128; 32%)		*P* value	
**Age (years)**								.85
	Mean (SD)	63.8 (13.1)		63.6 (10.7)				
	Median (IQR)	66 (56-73)		65 (59-71)				
	Minimum-maximum	19-88		20-84				
**Sex, n (%)**								.14
	Female	155 (57.8)		64 (50)				
	Male	113 (42.2)		64 (50)				
**Race, n (%)**								.78
	White	235 (87.7)		114 (89.1)				
	Black	25 (9.3)		11 (8.6)				
	Other	4 (1.5)		3 (2.3)				
	Unknown or declined	4 (1.5)		1 (0.78)				
**Risk level, n (%)**								.69
	Low	224 (83.6)		109 (85.2)				
	Intermediate	40 (14.9)		16 (12.5)				
	High	4 (1.5)		3 (2.3)				
**Procedure, n (%)**								.99
	Major abdominal	31 (11.6)		15 (11.7)				
	Spine	69 (25.7)		35 (27.3)				
	TJR^a^ hip	75 (28)		36 (28.1)				
	TJR knee	93 (34.7)		42 (32.8)				

^a^TJR: total joint replacement.

**Table 4:** Comparison of secondary end points between non-Pip and Pip patients using marginal structural models with stabilized inverse probability of treatment weighting (SIPTW).

Secondary end points		Non-Pip (n=268; 68%)	Pip (n=128; 32%)	*P* value	
**Length of stay (days)**					.009
	Mean (SD)	3.1 (2.8)	2.4 (2.4)		
	Median (IQR)	2.9 (1.1-3.9)	1.9 (1.0-3.1)		
	Minimum-maximum	0-27.8	0-14.2		
	Mean ratio (95% CI)	Reference	0.76 (0.62-0.93)		
**7-day readmission**					.38
	Patients, n (%)	9 (3.4)	2 (1.7)		
	Relative risk (95% CI)	Reference	0.51 (0.11-2.31)		
**30-day readmission**					.73
	Patients, n (%)	13 (4.9)	5 (4.1)		
	Relative risk (95% CI)	Reference	0.83 (0.30-2.31)		
**30-day emergency department return**					.85
	Patients, n (%)	26 (9.7)	13 (10.3)		
	Relative risk (95% CI)	Reference	1.06 (0.56-2.01)		

## Discussion

### Primary Result and Comparison With Previous Work

Our results demonstrate that Pip, a novel mobile DHP that combines both human HCs and technology, is feasible to use to engage patients during their perioperative journey. Pip engagement was also associated with reduced hospital LOS. Of the patients who attended ≥1 HC session, over two-thirds completed the program, which is far better than industry benchmarks. As the mean age of our Pip cohort was 63 (range 20-84) years, this demonstrates great engagement and feasibility across many ages.

There are multiple DHPs that have been developed in recent years, some focusing on chronic medical conditions or symptom monitoring [8,22]. There are also other mobile DHPs that are designed to provide enhanced recovery after surgery protocol guidance, presurgical instructions, and patient adherence to said protocols to help improve outcomes for both patient and hospital [13,23-25]. However, this is one of the first perioperative DHPs to involve a human-HC interaction to help allay patients’ anxiety, alleviate clinical and administrative burden, and digitize perioperative protocols and instructions, not only through the preoperative period but also in the postoperative setting.

Our primary outcomes focused on human-technology and human-human engagement [26], that is, how the user interacts with both the technology and the emotional response to the human interaction. The excellent short-term user experience patient satisfaction scores and net promotor scores demonstrate high patient satisfaction and the commercializability of the product. Over 95% (93/95) of patients were satisfied with Pip and would recommend it. Pip generated impressive patient satisfaction scores when discussing patient-HC interaction, and nearly all respondents agreed that they would refer a friend or colleague going through surgery to use Pip. Furthermore, the patient capture rate and DHP use are far in excess of industry benchmarks. As 70% (97/138) of the patients who proceeded to surgery completed the program, this is also an advantage to health systems for cost reduction with shorter LOS and decreased complications with improved optimization.

Positive comments on the use of Pip in the perioperative journey supported that both the HC and the DHP, in concert with the HC, helped to relieve anxiety, hold patients accountable using encouragement, and empower patients to take responsibility for their care. These comments reflect observations that high-level engagement, representing a partnership for shared leadership, is very important [27]. Furthermore, the patients’ desire to be not only heard (as a token of involvement) but listened to (reflecting a deeper conversation addressing the core issues at the center of the patient’s thoughts) is essential to continued patient engagement with the platform [28].

An interesting finding was the significant LOS reduction with Pip use. While impressive, we acknowledge that this is a small cohort and the study was not powered for this outcome. Nevertheless, using a marginal structural model with SIPTW, the sample size is preserved close to the original data and produces an appropriate estimation of the main intervention effect while maintaining an appropriate type I error rate. Pip was significantly associated with a 24% reduction in postoperative LOS (mean ratio 0.76; 95% CI 0.62-0.93), and Pip was associated with 49% and 17% lower risk in readmissions at 7 and 30 days (though not statistically significant). Though we have matched for age, procedure, and perioperative risk score and feel that the likely effect is the Pip intervention, further studies powered for these outcomes are necessary. As all patients in the Pip and standard of care cohorts attended our perioperative risk stratification and optimization clinic, these findings may be even more pronounced when a robust perioperative clinic is not readily available in smaller health systems or when patients have limited access to care. Furthermore, we feel that the personalized contact from the HC offers advantages over other content-only DHPs. The DHP and HC combination, personalized optimization protocols, and high satisfaction correlate with positive patient outcomes. Further studies examining the type and frequency of Pip DHP or HC interaction based on patient comorbidity would offer interesting insights into more widespread deployment of the DHP and HC resources for those most likely to benefit.

### Limitations

This study has several limitations. Because all patients were computer literate and had ready access to smartphones, there may have been a participation bias that influenced this feasibility study. Adding a web-based interface or the ability to add a caregiver could reduce the technological barrier for some patients. Additionally, those patients who chose to participate in Pip may have contributed to selection bias for patients who are more motivated to optimize before surgery. Our patient satisfaction data are limited to descriptive statistics, and further information will be collected in the future. Additionally, the satisfaction question assessing the HC was framed positively, and this may have skewed the patient rating. Regarding secondary end points, while the LOS reduction is significant and the readmission rate reduction trends reasonably, this study was neither designed nor powered for these outcomes. Additionally, because many referrals took place within the health system network, there may have been other factors contributing to these outcomes. Further study is required with a larger cohort designed to examine both clinical outcomes and PROs.

### Conclusions

In summary, Pip is a novel mobile health care digital platform that combines human HCs and preoperative optimization content that is feasible to engage surgical patients during their perioperative journey, with high patient enrollment and very high engagement with the HCs. Patient satisfaction was high for those participating in Pip. When compared to a similar cohort without Pip, surgical patients that participated in Pip experienced a reduced LOS in our feasibility study. Further studies are required to better assess the clinical and PRO impacts of the use of Pip or similar DHPs combined with HCs during the perioperative journey, as the use of an HC may offer improved patient-centered outcomes.

## Appendix

Multimedia Appendix 1 Baseline characteristics and outcomes before weighting by Pip versus non-Pip.

## Abbreviations

DHP: digital health platform
ED: emergency department
HC: health coach
HIPAA: Health Insurance Portability and Accountability Act
LOS: length of stay
PRO: patient-reported outcome
RR: relative risk
SIPTW: stabilized inverse probability of treatment weighting
