# Supplementary material for: A Novel Digital Health Platform With Health Coaches to Optimize Surgical Patients: Feasibility Study at a Large Academic Health System
Source: JMIR Perioper Med. 2024 Apr 4;7:e52125. doi: 10.2196/52125 (PMC11027047; doi:10.2196/52125)
Supplement: Multimedia Appendix 1 [file periop_v7i1e52125_app1.docx]

**Table S1.** Baseline characteristics and outcomes before weighting by Pip versus non-Pip. SD=Standard deviation. Min=Minimum. Max=Maximum

| *Variable* | *All n=396* | *Non-Pip n=268 (68%)* | *Pip n=128 (32%)* | *p-value* |
| --- | --- | --- | --- | --- |
| *Age* | *n=396* | *n=268* | *n=128* | *0.016* |
| Mean(SD) | 63.9 (12.4) | 64.7 (12.8) | 62.2 (11.3) |  |
| Median(25th:75th) | 66.0 (57.0 : 73.0) | 67.0 (57.0 : 74.0) | 63.5 (56.0 : 69.5) |  |
| Min:Max | 19.0 : 88.0 | 19.0 : 88.0 | 20.0 : 84.0 |  |
| *Sex* | *n=396* | *n=268* | *n=128* | *0.19* |
| Female | 220 (56%) | 155 (58%) | 65 (51%) |  |
| Male | 176 (44%) | 113 (42%) | 63 (49%) |  |
| *Race* | *n=396* | *n=268* | *n=128* | *0.84* |
| White | 347 (88%) | 234 (87%) | 113 (88%) |  |
| Black | 37 (9%) | 26 (10%) | 11 (9%) |  |
| Other | 7 (2%) | 4 (1%) | 3 (2%) |  |
| Unknown/Declined | 5 (1%) | 4 (1%) | 1 (1%) |  |
| *Risk Level* | *n=396* | *n=268* | *n=128* | *0.14* |
| Low | 332 (84%) | 218 (81%) | 114 (89%) |  |
| Intermediate | 58 (15%) | 45 (17%) | 13 (10%) |  |
| High | 6 (2%) | 5 (2%) | 1 (1%) |  |
| *Risk Value*100* | *n=396* | *n=268* | *n=128* | *0.032* |
| Mean(SD) | 1.2 (1.5) | 1.3 (1.6) | 1.0 (1.3) |  |
| Median(25th:75th) | 0.7 (0.4 : 1.4) | 0.7 (0.4 : 1.5) | 0.5 (0.4 : 1.3) |  |
| Min:Max | 0.1 : 11.7 | 0.1 : 11.7 | 0.1 : 11.7 |  |
| *Procedure* | *n=396* | *n=268* | *n=128* | *0.26* |
| Major Abdominal | 46 (12%) | 34 (13%) | 12 (9%) |  |
| Spine | 101 (26%) | 61 (23%) | 40 (31%) |  |
